# Outcome of Endoscopic Resection of Rectal Neuroendocrine Tumors ≤ 10 mm

**DOI:** 10.3390/diagnostics14141484

**Published:** 2024-07-11

**Authors:** Roberta Elisa Rossi, Maria Terrin, Silvia Carrara, Roberta Maselli, Alexia Francesca Bertuzzi, Silvia Uccella, Andrea Gerardo Antonio Lania, Alessandro Zerbi, Cesare Hassan, Alessandro Repici

**Affiliations:** 1 Gastroenterology and Endoscopy Unit, IRCCS Humanitas Research Hospital, Via Manzoni 56, 20089 Rozzano, Italy; 2 Department of Biomedical Sciences, Humanitas University, Via Rita Levi Montalcini 4, 20072 Pieve Emanuele, Italy; 3 Hematology and Oncology, IRCCS Humanitas Research Hospital, Via Manzoni 56, 20089 Rozzano, Italy; 4 Pathology Service, IRCCS, Humanitas Research Hospital, Via Manzoni 56, 20089 Rozzano, Italy; 5 Endocrinology and Diabetology Unit, IRCCS Humanitas Research Hospital, Via Manzoni 56, 20089 Rozzano, Italy; 6 Pancreatic Surgery Unit, IRCCS Humanitas Research Hospital, Via Manzoni 56, 20089 Rozzano, Italy

**Keywords:** endoscopic resection, endoscopic mucosal resection, endoscopic submucosal dissection rectal, neuroendocrine, neoplasm, tumor, transanal microsurgery, ultrasound endoscopy

## Abstract

Background and aim: Guidelines suggest endoscopic resection for rectal neuroendocrine tumors (rNETs) < 10 mm, but the most appropriate resection technique is unclear. In real-life clinical practice, the endoscopic removal of unrecognized rNETs can take place with “simple” techniques and without preliminary staging. The aim of the current study is to report our own experience at a referral center for both neuroendocrine neoplasms and endoscopy. Methods: Retrospective analyses of polypectomies were performed at the Humanitas Research Hospital for rNETs (already diagnosed or previously unrecognized). Results: A total of 19 patients were included, with a median lesion size of 5 mm (range 3–10 mm). Only five lesions were suspected as NETs before removal and underwent endoscopic ultrasound (EUS) before resection, being removed with advanced endoscopic techniques. Unsuspected rNETs were removed by cold polypectomy in eleven cases, EMR in two, and biopsy forceps in one. When described, the margins were negative in four cases, positive in four (R1), and indeterminate in one. The median follow-up was 40 months. A 10 mm polypoid lesion removed with cold snare polypectomy (G2 R1) needed subsequent surgery. Eighteen patients underwent EUS after a median time of 6.5 months from resection. The EUS identified local recurrence after 14 months in a 7 mm polypoid lesion removed with cold snare polypectomy (G1 R1); the lesion was treated with cap-assisted EMR. For all the other lesions, the follow-up was negative. Conclusions: When rNETs are improperly removed without prior staging, caution must be exercised. The data from our cohort suggest that even if inappropriate resection had happened, patients may be safely managed with early EUS evaluation.

## 1. Introduction

Neuroendocrine neoplasms (NENs) are heterogeneous tumors, ranging from indolent (well-differentiated neuroendocrine tumors, NETs) to aggressive forms, which are more biologically and clinically similar to their exocrine counterpart (poorly differentiated neuroendocrine carcinomas, NECs) [1]. The risk of harboring a metastatic disease ranges from 3 to 60%, depending on the NEN type, grade, and stage [2]. The gastrointestinal tract, together with the lungs, is a frequent site of origin of NENs, with the small intestine ranking first and the rectum second in terms of frequency [3], with the latter representing 12–27% of all gastrointestinal NENs [4,5].

Most rectal NENs (up to 90%) are, in fact, NETs, and their diagnosis is usually incidental, as the occurrence of symptoms, such as anal pain or discomfort, rectal bleeding, or obstruction, is rare [6].

Among all rectal tumors, 1–2% are estimated to be NETs [4,5], but an increasing incidence has been reported over recent decades, likely as a consequence of the progressive spread of screening colonoscopies [3,7,8,9,10].

rNET’s estimated prevalence is approximately 0.05% [11,12], and in the United States, the incidence rate reaches approximately 1.1 per 100,000 people, with a 10-fold increase between 1970 and the 2000s [9].

rNETs usually appear as small (<10 mm), roundish, and yellowish polypoid lesions, coated with normal mucosa (pit pattern type I according to the Kudo classification), while the typical “doughnut” appearance is usually seen in lesions > 10 mm [13,14]. They are generally located at the medial-inferior portion of the rectum (4 to 10 cm from the anal verge) [15]. Less-frequent endoscopic appearances include the semi-pedunculated shape, hyperemia, central depression, erosion, or ulceration, especially in lesions > 5 mm [15]. They usually involve the mucosal and submucosal layers, without the involvement of *muscularis propria* [16].

In terms of treatment, available guidelines suggest the use of modified endoscopic mucosal resection (EMR) for ≤10 mm rNETs, particularly EMR performed with a suction cap, which is able to reach an en bloc resection success rate of nearly 100%, endoscopic submucosal dissection (ESD), or endoscopic full thickness resection (eFTR) [17,18,19]. Conversely, standard polypectomy (with both hot or cold snare) and conventional EMR are thought to not guarantee a sufficiently complete resection rate of the lesion margins; thus, these techniques are no longer recommended [18,19,20].

The standardized use of endoscopic ultrasound (EUS) before resection in small lesions (<10 mm) is controversial but usually suggested to exclude the occurrence of lymphatic invasion and to evaluate the depth of invasions to plan the most feasible and optimal endoscopic resection [19,21], although its lower accuracy for small tumors should be considered [22,23].

In real-life clinical practice, the endoscopic removal of unrecognized rNETs frequently takes place with “simple” techniques and without preliminary imaging staging, as their macroscopic appearance resembles that of hyperplastic or adenomatous polyps and the endoscopist might fail to recognize them upfront [24]. As a matter of fact, only 18% of NET lesions were previously suspected as neuroendocrine [25]. On the other hand, rNETs correctly identified before endoscopic removal have higher complete resection rates compared to tumors resected as ordinary colonic polyps [26]. Therefore, if the management of correctly classified rNETs can be complex, that of unrecognized rNETs is even more challenging.

Based on these observations, the aim of the current study is to describe a real-life cohort of patients followed at a referral center for both endoscopy and NEN management, with a specific focus on patients who received a diagnosis of rNETs only after endoscopic resection.

## 2. Methods

This is a retrospective case series. We collected data on all consecutive patients with endoscopically resected rNETs followed at the IRRCS Humanitas Research Hospital, Rozzano, Milan, Italy, between 2014 and 2023.

Patients with a rectal lesion endoscopically resected, diagnosed as NET before or after resection, were included. The exclusion criteria included age < 18 years old; incomplete clinical data; insufficient histopathology data to confirm the diagnosis of rNET; absence of adequate follow-up (namely, patients were included if, after resection, they underwent at least one evaluation among simple endoscopy, EUS, or imaging).

Informed consent was waived, given the use of retrospective historic de-identified data.

Data on patients’ general characteristics, indications for endoscopy, lesions’ endoscopic features (including tumor location, size, morphology, and surface color), endoscopic technique, complications (such as bleeding and perforation), histology, outcomes, and follow-up modalities were collected from the electronic medical records and revision of procedures images.

Two subgroups were considered: patients with a previous known or suspected diagnosis of rNET and patients in whom rNETs were removed without awareness of their nature. In the first subgroup, patients usually presented with lesions with “typical” features of rNET; they frequently underwent a preliminary diagnostic biopsy and/or received previous rectal EUS evaluation. The subsequent endoscopic resection was planned in a dedicated setting and performed as per guidelines. In the latter subgroup, rNETs did not present with features suggestive of a possible neuroendocrine nature; therefore, these lesions were not recognized by the endoscopist and were removed based on the operator’s preference, usually with simple endoscopic techniques; the actual diagnosis was provided by histology thereafter.

The endoscopic resection techniques included:

- Cold-forceps polypectomy.
- Standard cold polypectomy.
- Traditional endoscopic submucosal resection (EMR), i.e., resection by snare electrosurgery (hot snare) with previous injection of a solution into the submucosal space to separate a mucosal lesion from the underlying *muscularis propria* to reduce the risk of thermal or mechanical injury to the underlying *muscularis propria*. Injection of submucosa is performed with adrenaline and methylene blue diluted in saline solution.
- cap-assisted EMR: EMR performed with adjunct use of transparent plastic cap (Olympus, straight, 12.4–14 mm–Olympus, Tokyo, Japan; or US Endoscopy, straight, 12.6–13.2 mm–US Endoscopy, Mentor, OH, USA), positioned extending approximately 3–4 mm beyond the distal end of the endoscope, in order to enhance lesion lifting using suction inside the cap and subsequent hot snare polypectomy.
- Endoscopic submucosal dissection (ESD), i.e., removal of the lesion dissecting the submucosa using a dedicated endoscopic through-the-scope needle-type knife with previous submucosal injection of colloid/crystalloid solution and dye [27,28,29].

“Simple” endoscopic treatments included forceps polypectomy or cold snare polypectomy, whereas “advanced” techniques included EMR and/or ESD.

All the “advanced” endoscopic resection techniques were performed at IRCCS Humanitas Research Hospital, Rozzano, Milan, Italy by expert endoscopists (i.e., >5 years of practice) using Fujifilm instruments (Fujifilm Corporation, Tokyo, Japan) and processor (600 and ELUXEO^®^ 700 series, ELUXEO video processor VP-7000, ELUXEO BL-7000 light source) and stiff open 10–15 mm hot resection snare (Boston Scientific Captivator, Marlborough, MA, USA) or a hybrid knife (Erbe Elektromedizin GmbH, Tübingen, Germany).

All preliminary and surveillance EUS examinations were performed at IRCCS Humanitas Research Hospital, Rozzano, Milan, Italy by expert endosonographers (i.e., >5 years of practice) using the Olympus GF-UCT180 series linear array echo-endoscope (Olympus Europa SE & CO. KG, Hamburg, Germany) combined with the new EU-ME2 echo-processor (Olympus SE & CO. KG, Hamburg, Germany).

Tumors were classified as G1, G2, or G3 lesions according to the WHO 2010 or 2019 classification of digestive system tumors based on the proliferation index (mitotic count and Ki67-related proliferation index) [30].

All the cases were discussed at the multidisciplinary NEN meeting at our European Neuroendocrine Tumor Society (ENETS) Center of Excellence (CoE).

The follow-up plan was based on the characteristics of the lesion and the state of the margins [2]. Patients diagnosed with rNETs before endoscopic resection that were properly removed were generally followed up with endoscopies every 6–12 months, including a rectal EUS generally performed after 6–12 months. Conversely, for patients whose tumor was not diagnosed before resection, a rectal EUS was generally planned 3–6 months after. Conventional radiological imaging (i.e., CT or MRI) as well as functional imaging were planned only on the suspicion of recurrence.

## 3. Results

We included 19 patients (M:F 12:7, median age 54 years, range 27–70 years) with a total of 19 rectal lesions. Twelve patients underwent colonoscopy for screening indication, seven patients because of gastro-intestinal symptoms (including abdominal pain, diarrhea, or bleeding).

**Figure 1:**
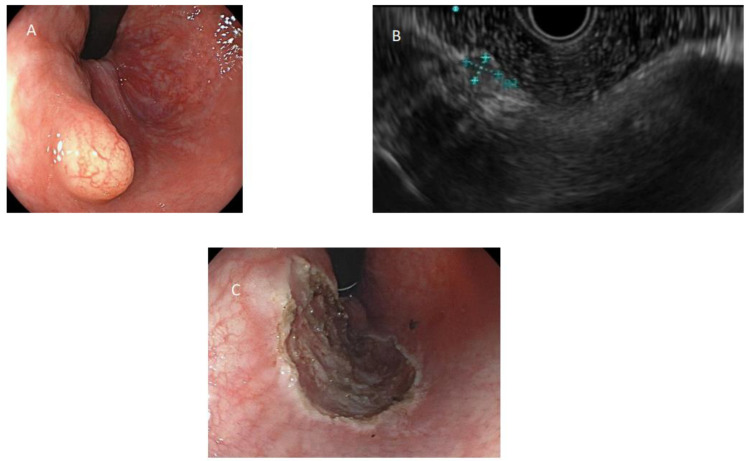
(**A**) Rectal neuroendocrine tumor with typical appearance of a yellowish sub-epithelial lesion, visualized in retroversion; (**B**) endoscopic ultrasound aspect of a rectal neuroendocrine tumor; (**C**) cutting eschar after removal using cap-assisted endoscopic mucosal resection.

**Figure 2:**
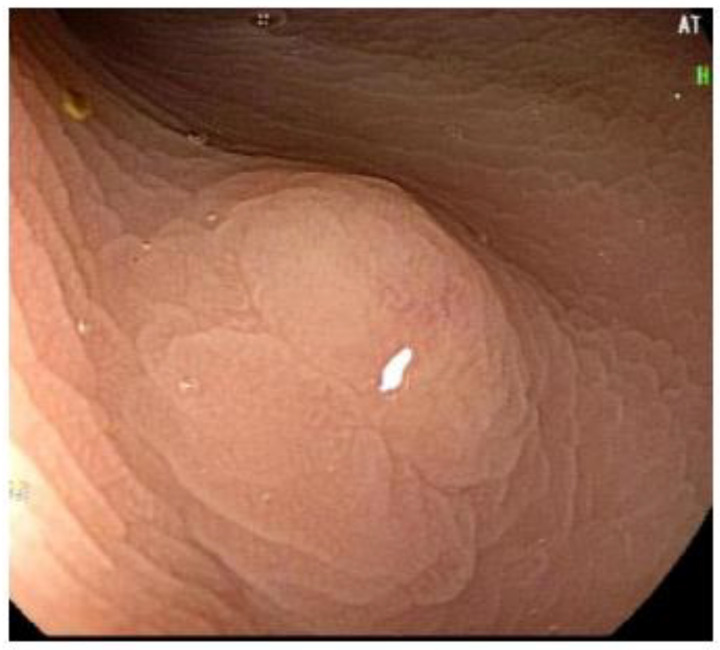
Rectal neuroendocrine tumor with less typical appearance, sessile polypoid.

The lesions were described as sessile polyps in eleven cases, semi-peduncolated in one, and sub-epithelial in seven cases, with a median size of 5 mm (range 3–10 mm). The median distance from the anal verge was 6 cm (range 3–10 cm). All sub-epithelial lesions (SELs) were described as yellowish, and two were additionally described as stiff-elastic. Endoscopic and EUS appearances of rNETs are represented in Figure 1 and Figure 2.

Only five lesions were correctly suspected to be NETs during index colonoscopy, based on their macroscopic characteristics; all were described as SELs, yellowish, covered by normal-appearing mucosa. All five lesions underwent EUS before resection, revealing hypoecoic lesions originating in the submucosa (#2) or in the mucosa (#3), with two lesions described as hypervascular. No lymph node involvement was reported, and all EUS reports suggested neuroendocrine lesions. Three lesions were also subjected to biopsy (forceps), confirming their neuroendocrine nature.

These lesions were subsequently removed by ESD in one case (8 mm lesion with submucosal origin) and cap-assisted EMR in the other four cases. At histology, four lesions were classified as G1 (Ki67 proliferation index, PI: 1% in three patients and 2,4% in one patient) and one as G2 (Ki67 4% PI). Resection margins were all described as negative. For all the lesions, the follow-up was negative (median follow-up time: 48 months, range: 5–81 months).

In the other 14 patients, the diagnosis of NET was made only after endoscopic resection. These lesions were removed by cold polypectomy in eleven cases, by EMR in two cases, and by avulsion with biopsy forceps in one case. At histology, eleven lesions were classified as G1 and three as G2 (Ki67 PI 4%, 6%, and 5%, respectively). When resection margins were described, they were negative in four cases, positive in four (i.e., R1), and indeterminate in one. In five cases, the margins were not described by the pathologist. The median follow-up time was 40 months (range 5–138 months).

A 10 mm polypoid lesion removed with cold snare polypectomy (G2, Ki67 PI 6%, R1) needed immediate subsequent surgical resection with lymphadenectomy (six of the sixteen lymph nodes that were removed were positive).

Local recurrence occurred after 14 months in a 7 mm polypoid lesion removed with cold snare polypectomy (G1, Ki67 PI 2%, R1), which was then treated with cap-assisted EMR with a negative subsequent 50-month follow-up.

For all the other lesions, including two lesions that resulted in R1 and lesions with missing data about margin status, the subsequent follow-up was negative.

Overall, 18 patients underwent subsequent EUS evaluation, with a median time of first evaluation from the endoscopic removal of 6.5 months (range 2–130 months). In seven cases, the EUS evaluation was within normal limits; in ten cases, the regular scarring results of the endoscopic removal were visible, while in one case, only the EUS evaluation performed 12 months after the first procedure highlighted the persistence of the disease, which was then re-treated with cap-EMR as previously reported. The subsequent EUS examinations were all negative.

In 10 patients, the rectal EUS was also repeated during the follow-up every 6–12 months after the resection.

Table 1 and Table 2 summarize the clinical characteristics of the 19 included patients. 

## 4. Discussion

The incidence of rNETs has significantly increased in recent years, likely due to the widespread adoption of endoscopic colorectal cancer screening programs [3,8].

This represents a challenge in clinical practice, as the majority of small rNETs are not correctly recognized and classified before endoscopic resection [25]. Consequently, they are often removed without an accurate a priori assessment of the metastatic risk and with the use of standard endoscopic techniques not recommended by the available guidelines.

Given their small size and non-specific macroscopic characteristics (i.e., the round shape, the yellowish color), it is difficult to distinguish rNETs from colonic diminutive polyps, both hyperplastic and adenomatous [24]. Therefore, efforts should be made to improve the prompt recognition of rNETs by endoscopists.

The adjunct use of virtual-assisted techniques is promising, but both virtual chromoendoscopy and artificial intelligence (AI) are still of limited help in distinguishing luminal NENs. Narrow band imaging (NBI) is described as helpful only in recognizing invasive rNETs, when the absence of a pit pattern with large amorphous areas (Kudo V) is detected [14]. AI is rapidly advancing, but for diminutive polyps, it is mainly effective in distinguishing between adenomatous and non-adenomatous lesions [31,32]. However, given the increase in the incidence of these tumors, it is likely that in the near future, AI will be able to provide concrete help.

Incorrect rNET removal can be problematic, particularly for the subsequent management, especially considering that even for lesions following the correct method, existing guidelines are sometimes unclear and not fully evidence-based [18].

Nevertheless, data from our case series, although limited by the small sample size and retrospective nature, seem reassuring, partially contrasting with some more alarming literature results, for both the completeness of resection and outcomes [18,26]. In particular, in the study by Fine et al., out of 190 unsuspected rNETs subjected to attempted primary resection at index colonoscopy, 148 underwent standard polypectomy; the overall successful R0 resection rate was only 17% [25]. Moreover, a Chinese study identified both the use of simple polypectomy and treatment during index colonoscopy as risk factors for incomplete resection in a cohort of patients with small (≤10 mm) rNETs; treatment in the early period was also a significant predisposing factor for inappropriate choice of polypectomy [33].

Since the prognosis of rNETs is usually attributed to the successful complete resection of the lesion, salvage therapies such as EMR, ESD, or transanal endoscopic microsurgery are suggested when complete resection is not achieved [34]. A French study suggested systematically applying resection of the visible scar after an incomplete endoscopic resection [35].

However, it is worth noting that for small G1 lesions, incomplete resection alone may not significantly impact the patient’s prognosis. In fact, in the same Chinese study, among 83 patients with incomplete resection, 18 underwent salvage treatment, while 65 refused it. However, patients who were only in follow-up showed no recurrence of the disease [33]. Similar data also emerged from other case series comparing endoscopic treatment with surgical treatment, where surgical treatment guarantees almost zero rates of incomplete resection, but the overall prognosis does not appear to vary [36].

These results are certainly limited by the retrospective nature of the available studies, with subsequent short follow-up times, whereas in some series, the rare appearance of distant metastases occurred many years after the first endoscopic intervention [37]. Furthermore, resection of small lesions with “simple” techniques makes margin assessment particularly difficult, thus increasing the rate of R1 or indefinite histopathological definitions [18].

For lesions <1 cm with R1 after correct endoscopic removal, current ENETS guidelines suggest a second endoscopic resection (or transanal minimally invasive surgery) in order to achieve R0 or, alternatively, a watch-and-wait approach if EUS, MRI, and repeat biopsies are negative (level of evidence 3 and grade of recommendation C) [18]. However, as mentioned above, it is still unclear whether R1 accurately predicts the presence of residual tumor at the resection site and, even in that case, if the presence of residual tumor impacts the patient’s prognosis, posing the problem that further resection could result in overtreatment.

In our cohort, despite the high number of R1/Rx resections, results are overall favorable for G1 lesions, without reported disease progression, and without the need for subsequent surgery. However, all the rNETs included in the current series were well differentiated, low- to moderate-grade tumors, which is in line with data from the literature [33], even though small lesions can also show a more aggressive behavior [38]. In fact, in one case, surgery was required after endoscopic R1 resection, as the lesion was a G2 with evidence of lymph node metastases (so the need for surgery was not related to margin status).

Anyway, it is worth noting that in our cohort, among the 14 patients with a diagnosis of NET made after endoscopic resection, all of them underwent subsequent EUS evaluation after a median interval time of 6,5 months, which proved to be accurate in both the detection of recurrent disease (in one lesion after 14 months from resection, which was successfully re-treated with cap-EMR) and in the identification of lymph node metastases in a lesion that required subsequent surgery. Moreover, it might be reasonable to proceed with caution and apply all the available instruments to ensure a complete resection when needed, considering that, even if R1 is reported to have a generally good prognosis, the available studies are limited by the short follow-up time, and that a second endoscopic resection is a safe procedure, especially in comparison with surgical resection (also taking into account the delicate area of the rectum).

Furthermore, rectal EUS, in comparison with EUS in other areas, is less invasive, as it requires simple preparation (enema) and does not require sedation. Therefore, even if the current guidelines do not encourage a wide use of rectal EUS [39], we believe that after balancing costs and benefits, rectal EUS should be performed after an incidental endoscopic resection of a rNET. The execution of a subsequent EUS represents a modality of surveillance, but also a complementary tool to the histology.

However, it is important to highlight that in clinical practice, distinguishing between the scar (scar tissue) and residual tumor after resection can be challenging. While the scar usually appears as a thickening of the mucosa and/or submucosa, with ill-defined borders and the presence of irregular hypo-echoes, the residual tumor has the same characteristics as the primary lesion, particularly with the presence of a defined lesion with vascularization.

The limited use of MRI and functional imaging, which are currently considered necessary in follow-up by guidelines to complete the evaluation after R1 resection or if the resected lesion is G2-G3 or with additional risk factors (such as lympho-vascular invasion) [18], could be questioned. In our cohort, however, the use of these techniques was generally tailored to each single patient and reserved only for cases of suspected recurrence, and unfortunately, in some cases, data were missing (which is obviously a limitation due to the retrospective study design). It should be noted, however, that not only did previous guidelines not mention the routine use of these methods [19], but that even in the current ones, the strength of recommendations and quality of evidence are considered to be low [18]. Moreover, in contrast to the work of O’Neill et al., which suggests that even small rNETs frequently present with metastases at diagnosis (which are detected by MRI and PET scan) [40], in our experience, not only were recurrences detected by EUS alone, but the frequency of lymph node metastases at diagnosis was also very low, and EUS was able to identify all of them, whereas during follow-up, neither MRI nor PET provided further relevant findings.

Of course, the favorable outcomes of our cohort might be partially affected by the fact that our center is a referral center for both endoscopy and NEN management (with the availability of a dedicated multidisciplinary team), which is not a true representation of all clinical realities. In particular, the easy access to EUS performance by experts in the field represents, in our opinion, a key factor that is not everywhere available, which strengthens the importance of proper referral to tertiary centers for these specific tumors.

Finally, in our cohort, only one patient underwent ESD (an 8 mm tumor correctly suspected to be a rNET before endoscopic resection), so no observations can be made regarding this endoscopic technique. However, ESD has gained attention as a safe and effective procedure in rNETs [41].

## 5. Conclusions

When rNETs are improperly removed without prior imaging staging, caution should be exercised in the subsequent follow-up, as literature suggests that even small lesions may exhibit heterogeneous behavior, with local recurrence or nodal involvement [26,38].

**Figure 3:**
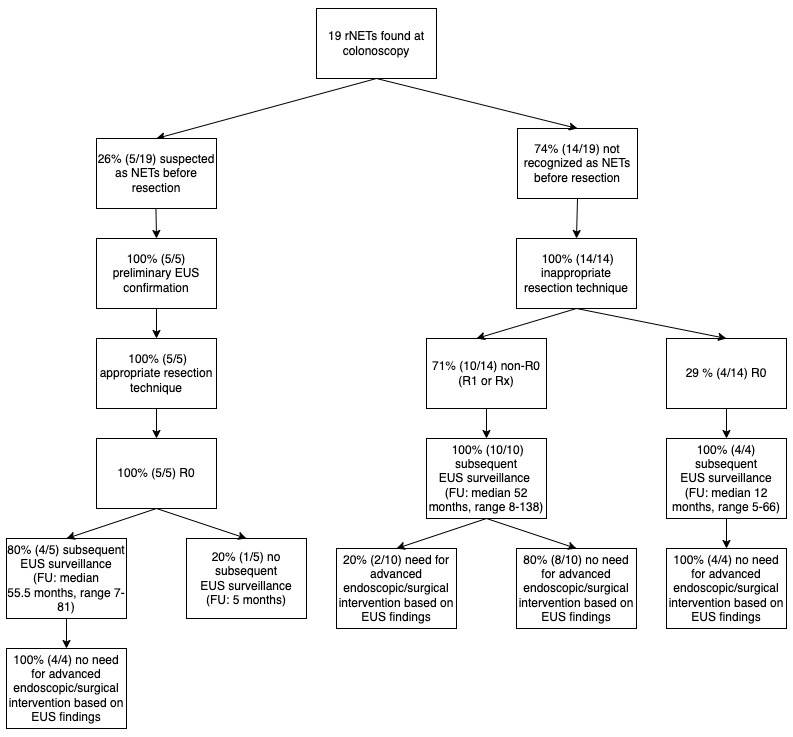
Flowchart showing how misrecognition and resection with inappropriate techniques led to a high percentage of non-R0 lesions and how surveillance with rectal endoscopic ultrasound (EUS) was fundamental in the subsequent clinical management.

Data from our cohort seem to suggest that even if inappropriate resection has happened and positive or doubt margins are found on the histopathological piece, patients may be safely managed with early EUS evaluation, excluding eventual nodal involvement and ensuring subsequent endoscopic resection if recurrence or residue on the site of removal is found (Figure 3). Even if these efforts produce an uncertain impact on prognosis, it is worth doing it in consideration of the relatively safe and less invasive approach as compared to the low but existing risk of a lesion with aggressive behavior and the subsequent risk of undergoing surgery.

A larger sample size is needed to draw more solid conclusions, although these preliminary results encourage the early referral to tertiary centers whenever a NET diagnosis is made. From a speculative point of view, efforts should be made to improve the prompt recognition of rNETs by endoscopists, and in this scenario, as a future perspective, AI might be of help.

## Figures and Tables

**Table 1:** Characteristics of included patients unsuspected as rectal neuroendocrine tumors before endoscopic resection.

**SEX**	female	male	male	male	male	female	male	male	male	female	male	female	female	female
**COLONOSCOPY INDICATION**	symptoms	screening	screening	symptoms	screening	symptoms	screening	symptoms	screening	screening	symptoms	screening	symptoms	screening
**AGE**	33	60	69	51	54	42	53	43	70	57	38	60	27	56
**DISTANCE FROM ANAL VERGE (CM)**	3	10	4	7	10	6		5		8	10	5	6	7
**MACROSCOPIC APPEARANCE**	polyp (sessile)	polyp (semiped)	polyp (sessile)	polyp (sessile)	polyp (sessile)	SEL	polyp (sessile)	polyp (sessile)	SEL	SEL	polyp (sessile)	polyp (sessile)	polyp (sessile)	SEL
**DIMENSION (MM)**	7	5		10	5	3	5	5	5	8	4	10	4	5
**EUS AT DIAGNOSIS**	no	no	no	no	no	no	no	no	no	no	no	no	no	no
**BIOPSY AT DIAGNOSIS**	no	no	no	no	no	yes	no	no	no	no	no	no	no	no
**HISTOLOGY**						G1, <2%								
**RESACTION TECHNIQUE**	cold polypectomy	cold polypectomy	cold polypectomy	cold polypectomy	EMR	forceps polypectomy	cold polypectomy	cold polypectomy	cold polypectomy	EMR	cold polypectomy	cold polypectomy	cold polypectomy	cold polypectomy
**COMPLICATIONS**	no	no	no	no	no	no	no	no	no	no	no	no	no	no
**HISTOLOGY**	G1, <1/10	G1, 0/10, 1%	G1	G2, 2/10, 6%	G1, 0/10, 1%	G1	G2, <2/10, 5%	G1, 1/10, 2%	G1, 0/10, 1%	G1,0/10, 1%	G1, 1%	G1, 1%	G2, 1/10, 2%	G1, 2/10, 1%
**MARGINS**	negative	not assessable		positive	negative		positive	positive (focal)		negative			negative	positive
**FU DURATIONS (MONTHS)**	12	99	132	17	66	29	12	50	75	5	138	8	6	8
**FU OUTCOMES**	neg	neg	neg	subsequent surgery (RAR + lymphadenectomy)	neg	neg	neg	recurrence after 14 months, treated with EMR cap-assisted (G1, 1/10, 2%), subsequent 12 month FU negative	neg	neg	neg	neg	neg	neg
**SURVEILLANCE WITH EUS**	yes	yes	yes	yes	yes	yes	yes	yes	yes	yes	yes	yes	yes	yes
**TI BETWEEN** **RESECTION AND EUS**		5		7	6	2	6	12	36	5	130	5	5	8
**REPEATED EUS**	yes	no	yes	no	no	yes	yes	yes	yes	yes	no	yes	yes	yes
**EUS FINDINGS**	normal	normal	scar	surgery outcome	scar	normal	wall thickening	recurrence	scar	scar	normal	scar	scar	scar
**OTHER FU IMAGING TOOLS**	endoscopy with biopsy	endoscopy with biopsy; MRI		MRI	endoscopy	PET		endoscopy	endoscopy with biopsy	endoscopy with biopsy	endoscopy with biopsy	endoscopy	endoscopy	endoscopy

EMR: endoscopic mucosal resection; EUS: endoscopic ultrasound; FU: follow-up; MRI: magnetic resonance imaging; PET: positron emission tomography; SEL: sub-epithelial lesion; TI: time interval.

**Table 2:** Characteristics of included patients suspected as rectal neuroendocrine tumors before endoscopic resection.

**SEX**	female	male	male	male	male
**COLONOSCOPY INDICATION**	symptoms	screening	screening	screening	screening
**AGE**	29	65	69	50	55
**DISTANCE FROM ANAL VERGE (CM)**	4	10	4	10	6
**MACROSCOPIC APPEARANCE**	SEL	SEL	SEL	SEL	SEL
**COLOR**	yellowish	yellowish	yellowish	yellowish	yellowish
**CONSISTENCY**	stiff-elastic			stiff-elastic	
**DIMENSION (MM)**	8	5	3	4	5
**EUS AT DIAGNOSIS**	yes	yes	yes	yes	yes
**ECOGENICITY**	hypo	hypo	hypo	hypo	hypo
**VASCULARIZATION**	hyper		hyper		
**LAYER OF ORIGIN**	submucosa	mucosa	submucosa	mucosa	mucosa
**LYMPHNODES**	no	no	no	no	no
**BIOPSY AT DIAGNOSIS**	no	no	yes	yes	yes
**HISTOLOGY**			G2, 4%	G1	G1, <3%
**RESACTION TECHNIQUE**	ESD	EMR cap-assisted	EMR cap-assisted	EMR cap-assisted	EMR cap-assisted
**COMPLICATIONS**	no	no	no	no	no
**HISTOLOGY**	G1, 1/10, 1%	G2, 3/10, 4%	G1, 1/10, 2,4%	G1, <1/10, <1%	G1, 1/0, 1%
**MARGINS**	negative	negative	negative	negative	negative
**FU DURATIONS (MONTHS)**	7	5	48	81	63
**FU OUTCOMES**	neg	neg	neg	neg	neg
**SURVEILLANCE WITH EUS**	yes	no	yes	yes	yes
**TI BETWEEN** **RESECTION AND EUS**	3		12	12	15
**REPEATED EUS**	no	no	yes	yes	yes
**EUS FINDINGS**	normal		normal	scar	scar
**OTHER FU IMAGING TOOLS**			endoscopy; MRI	endoscopy; MRI	MRI; PET

EMR: endoscopic mucosal resection; ESD: endoscopic submucosal dissection; EUS: endoscopic ultrasound; FU: follow-up; MRI: magnetic resonance imaging; PET: positron emission tomography; SEL: sub-epithelial lesion; TI: time interval.

## Data Availability

The original contributions presented in the study are included in the article, further inquiries can be directed to the corresponding author.
